# Growth of Hybrid Perovskite Crystals from CH_3_NH_3_PbI_3–*x*_Cl*_x_* Solutions Subjected to Constant Solvent Evaporation Rates

**DOI:** 10.3390/ma16072625

**Published:** 2023-03-26

**Authors:** Ioan Petrovai, Otto Todor-Boer, Leontin David, Ioan Botiz

**Affiliations:** 1 Faculty of Physics, Babes-Bolyai University, M. Kogalniceanu Str. 1, 400084 Cluj-Napoca, Romania; j.petrovai@gmail.com (I.P.); leontin.david@ubbcluj.ro (L.D.); 2 Interdisciplinary Research Institute in Bio-Nano-Sciences, Babes-Bolyai University, Treboniu Laurian 42, 400271 Cluj-Napoca, Romania; 3 INCDO-INOE 2000, Research Institute for Analytical Instrumentation, Donath Street 67, 400293 Cluj-Napoca, Romania; otto.todor@icia.ro

**Keywords:** perovskites, solvent evaporation, nucleation and growth, crystal dendrites

## Abstract

In this work, we subjected hybrid lead-mixed halide perovskite (CH_3_NH_3_PbI_3–*x*_Cl*_x_*) precursor inks to different solvent evaporation rates in order to facilitate the nucleation and growth of perovskite crystals. By controlling the temperature of perovskite solutions placed within open-air rings in precise volumes, we established control over the rate of solvent evaporation and, thus, over both the growth rate and the shape of perovskite crystals. Direct utilization of diluted lead-mixed halide perovskites solutions allowed us to control the nucleation and to favor the growth of only a low number of perovskite crystals. Such crystals exhibited a clear sixfold symmetry. While crystals formed at a lower range of temperatures (40–60 °C) exhibited a more compact dendritic shape, the crystals grown at a higher temperature range (80–110 °C) displayed a fractal dendritic morphology.

## 1. Introduction

In recent years, perovskite-based solar cells have reached a certified power conversion efficiency of up to 25.7% [1] due to the optimization of crystalline perovskite microstructure [2,3,4]. Moreover, by altering the crystallization kinetics of quasi-2D perovskites, the external quantum efficiency of light-emitting diodes based on such materials has been shown to exceed 18.15% [5,6,7]. Thus, in order to obtain the most suitable perovskite materials for various energy devices, it is important to control the microstructure of these materials, which is now possible in an ambient environment by utilizing simple deposition methods such as spin coating [8,9], drop casting [10,11,12] or dip coating [13,14,15]. Other scalable deposition methods used to fabricate perovskite active layers of specific crystalline microstructure include spray coating [16,17,18], slot-die coating [19], blade coating [20,21,22], D-bar coating [23,24] and various roll-to-roll processes [25]. However, such rapid deposition methods are not ideal to produce model perovskite structures that could be precisely evaluated with respect to their optoelectronic properties. Moreover, a slower method for the generation of perovskite-based microstructures seems to lead to thin crystalline films exhibiting higher quality and improved properties [26,27,28]. Although various perovskite processing procedures have been designed and developed [29,30,31,32], it is still challenging to fully understand and precisely control the mechanisms of solid-phase nucleation and crystal growth from various perovskite solutions. Nonetheless, this aspect is highly important because perovskite crystals have been shown to display important advantages, such as enhanced optoelectronic properties (high absorption coefficient, extended diffusion length, improved charge carrier mobility, etc.) and stability [33,34,35,36]. Such qualities make them highly suitable for applications like solar cells, light-emitting diodes, photodetectors, lasers, etc.

There are many different methods to obtain crystals of perovskites [37,38,39]. For instance, such methods may rely on cavitation-triggered asymmetrical crystallization [40] or on the utilization of space confinements [41]. While the former method leads to monocrystalline perovskite films ideal for solar cells [40], the latter provides important possibilities for mass production of ultrathin crystalline wafers [41]. Films comprised of perovskite crystals can also be obtained by employing the vapor-phase epitaxial growth [42] or surface-tension-assisted growth [43] methods. Other approaches to generate perovskite crystals are based on reduced solution temperature [44,45], inverse temperature crystallization [46,47,48], meniscus-assisted solution printing [49], anti-solvent vapor-assisted crystallization [34,50,51,52], solvent acidolysis crystallization [53], top-seeded solution growth under an ambient atmosphere [54] and many other methods. Resulting (organic–inorganic hybrid) perovskite crystals can be used for bandgap engineering [34]. Other efficient methods to obtain perovskite crystals may count on the utilization of polymers to control the nucleation process [55] or the use of single-walled carbon nanotube-based additives to retard crystal growth [56]. Furthermore, silk fibroin ca be employed as crystal growth template to align the perovskite crystals, thereby massively improving their electron mobility [57], while the usage of printed hydrophilic–hydrophobic substrates can lead to large-scale perovskite single-crystal arrays for photodetectors and other optoelectronic devices [58].

In this paper, we aim to establish a simple practical method to produce well-defined perovskite crystals that can be used in photovoltaic or emission energy devices. To do so, we utilize perovskite precursor ink solutions of precise volumes. The latter are placed into open-air Teflon rings and heated to a desired temperature, while kept under direct, real-time observation using an optical microscope (OM). By controlling the solution temperature, we dictate a specific yet constant solvent evaporation rate that leads to an increase in perovskite concentration over time, consequently initiating the nucleation and growth of lead-mixed halide perovskite crystals. By employing different solution temperatures, we further control not only the growth rate of perovskite crystals but also their shape and morphology.

## 2. Materials and Methods

I101 perovskite precursor ink was purchased from Ossila Ltd. (Sheffield, UK). Such systems have previously been used as efficient solar cell materials [59,60,61] and were produced by the manufacturer by dissolving methylammonium iodide (MAI) and lead chloride (PbCl_2_) in dimethylformamide (DMF) in a 3:1 ratio [62,63,64]. Further exposure to heat converted I101 perovskite to methylammonium lead halide perovskite (CH_3_NH_3_PbI_3–x_Cl_x_). Because MAI:PbCl_2_ was used as a precursor material, air processing in a low-humidity environment (20% to 35%) was possible.

**Figure 1:**
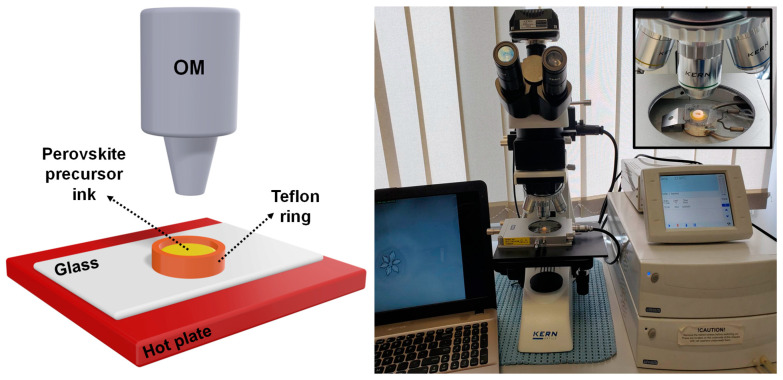
(**Left**) Schematic representation of the experimental setup used to monitor the growth of perovskite crystals in real time and direct space. (**Right**) An optical photograph depicting the equipment used to conduct the work presented herein. A hot stage connected to its corresponding temperature controller is positioned under the OM. The OM camera is linked to a PC. A sample (i.e., a Teflon ring glued on a glass substrate) is placed on the hot stage. The latter is more visible in the zoomed inset shown in the upper right corner.

The following experimental setup was used to study perovskite crystal formation. A Teflon ring with a 3 mm diameter and a 2 mm height was mounted and glued on a standard microscope glass slide. The setup was further treated in a UV ozone cleaner and placed on a Linkam THMS600 hot stage under an OM. The temperature of the hot stage could be adjusted and controlled with high precision between 40 °C and 110 °C (the hot stage is capable of regulating the temperature from 600 °C down to −196 °C when liquid nitrogen is utilized). Finally, 5 µL of the lead-mixed halide perovskite solution was injected in the Teflon rings and evenly spread over the glass substrate, leading to a solution of a thickness of about 700 µm (this value was deduced by considering that a 5 µL of perovskite solution represents a volume of about 5 mm^3^ and that the diameter of a used Teflon ring is 3 mm). A KERN OKN-177 OM with a 20× objective was used to observe the growth of perovskite crystals in real time and direct space (while maintaining the hot plate at a chosen constant temperature). The whole experimental setup is schematically represented in Figure 1. The solvent evaporation rate was mainly dictated by the processing temperature of the perovskite sample. Nonetheless, other physical parameters, such as the temperature of the perovskite solution and the room temperature and humidity, needed to be to controlled in order to perform comparable and reproducible experiments. To that end, the room temperature was regulated at 24 °C at all time via an air conditioning system, while the perovskite solutions were kept in the fridge at 5 °C prior to use.

A typical perovskite crystal growth experiment is described as follows. A cleaned Teflon ring glued on the glass substrate is positioned on the hot stage. The latter is then placed under the OM. Then, a specific sample processing temperature is set on the hot stage controller, while the OM objective is focused on the glass substrate. Once the substrate reaches the desired temperature, the perovskite solution is placed within the Teflon ring using a micropipette. The solution spreads instantly all over the surface. Then, the OM objective is slowly retracted to focus on the surface of the perovskite solution (or slightly within this solution yet in the vicinity of the surface). At this point, the homogeneous surface of the solution is continuously monitored. Generally, any inhomogeneity appearing after a certain time on the surface indicates the growth of a tiny perovskite crystal. Then, the objective of the OM is aligned so that the surface inhomogeneity is in the middle of the field of view and further focused so that the inhomogeneity is clearly visible. Furthermore, by taking advantage of the camera software installed on the PC, the camera can be programmed to record an optical micrograph (or a movie) at a specific distance in time. The collected micrographs are then analyzed and used to determine the growth rates of the corresponding crystal. Note that the OM objective that is chosen at the beginning of the experiment should be a low magnification (either 10× or 20×) to make it easier to find a tiny crystal/inhomogeneity. Later, when monitoring the growth of a given perovskite crystal, the objective can be switched to one capable of a higher magnification and better spatial resolution.

## 3. Results and Discussion

**Figure 2:**
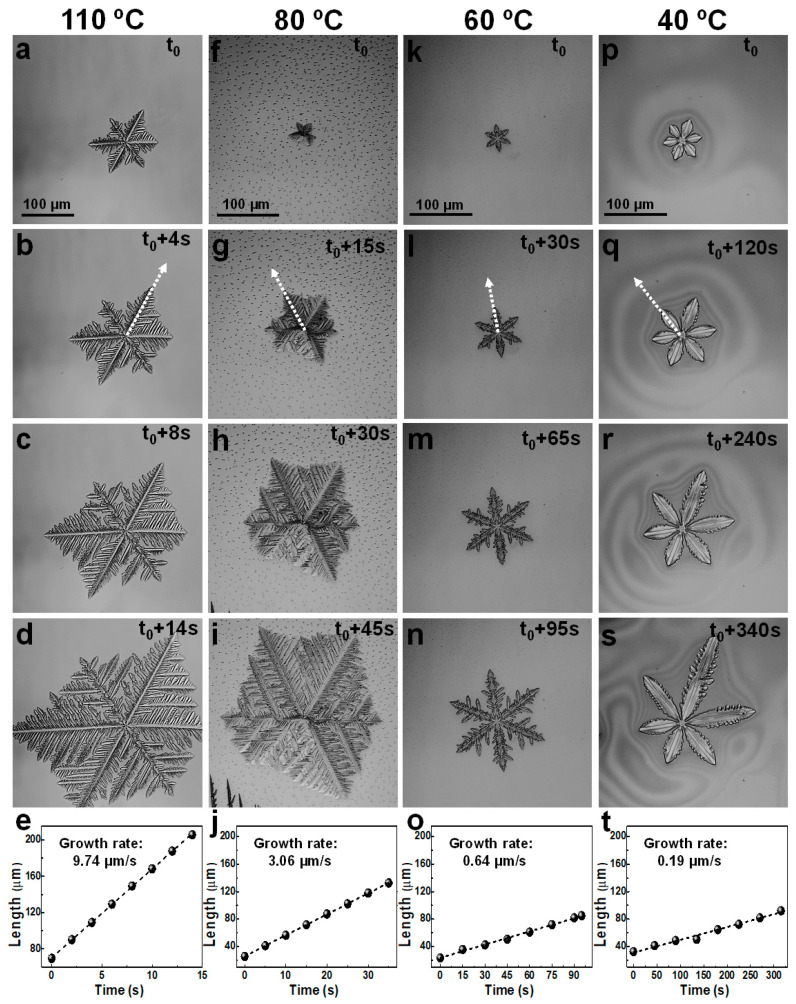
Optical micrographs depicting sixfold symmetrical perovskite crystals grown from a hybrid lead-mixed halide CH_3_NH_3_PbI_3–x_Cl_x_ perovskite precursor ink at processing temperatures of 110 °C (**a**–**d**), 80 °C (**f**–**i**), 60 °C (**k**–**n**) and 40 °C (**p**–**s**) and at corresponding growth rates of 9.74 µm/s (**e**), 3.06 µm/s (**j**), 0.64 µm/s (**o**) and 0.19 µm/s (**t**), respectively. The white dotted arrows indicate the direction along which the growth rates were determined. The dashed lines in (**e**,**j**,**o**,**t**) are linear fits to the data points. Their corresponding slopes indicate the average crystal growth rates. Note that t_0_ is different for each temperature.

Figure 2 shows optical micrographs of the CH_3_NH_3_PbI_3–*x*_Cl*_x_* hybrid crystals nucleated and grown at various temperatures from a lead-mixed halide perovskite precursor solution. At each selected processing temperature, our open-air experimental setup (see Figure 1) permitted us to maintain a corresponding solvent evaporation rate. The latter dictated not only the type of perovskite crystals that would be obtained but also how fast such crystals would grow, i.e., how flawless they would become from a structural point of view. We based our strategy on the well-known correlations between the evaporation and crystallization rates that occur when processing perovskite materials [65,66]. Although other variants of perovskite processing methods based on solvent evaporation were previously reported in the literature [67,68,69], the advantage of our setup lies in the possibility of nucleating only a small number of crystals, which can be grown independent of each other (note that perovskite crystals start “coalescing” only at very late stages of crystallization, when their size increases). As a result, hybrid CH_3_NH_3_PbI_3–*x*_Cl*_x_* perovskite crystals displaying a sixfold symmetry, represented by the six “tree” branches, nucleated and grew at a processing temperature of 110 °C (Figure 2a–d). Their rather high growth rate, determined to be over 9.7 µm/s (Figure 2e), favored the development of fractal dendrites, i.e., structures that exhibited a density that was substantially lower than that of an “ideal” faceted crystal [70,71]. Moreover, an almost hexagonal envelope formed from the six dendritic “trees” grown along the diagonal directions was observed. The crystal growth rates were generally determined (*i*) by measuring the distances from the center of a specific crystal to one if its growing tips/trees (the latter are indicated by dotted arrows in Figure 2) at different times after a given time (t_0_), (*ii*) by fitting the obtained data points with linear fits and (*iii*) by identifying the slopes of the linear fits as the corresponding average growth rates.

Furthermore, additional experiments performed at processing sample temperatures higher than 110 °C emphasized that under such conditions, the perovskite solutions were rather degrading, with some inhomogeneous (most often spherical) objects or structures appearing randomly all over the surface. Sometimes, crystals of ill-defined symmetry exhibiting a rather “melted/degraded” appearance were observed to form. More often, only some random objects, possibly irregular aggregates or very poor-quality crystalline structures, were observed on the surface after the total evaporation of the solvent.

At an even lower temperature of 80 °C, similar perovskite crystals also developed (Figure 2f–i). In this case, the only measurable difference was represented by a fewfold lower crystal growth rate (determined to be ~3 µm/s, Figure 2j). Otherwise, the fractal dendritic morphology was represented again by six dendritic “trees” forming a hexagonal envelope. When the temperature was lowered to 60 °C, thereby drastically reducing the solvent evaporation rate, perovskite crystals exhibiting a more compact (i.e., closer to the density of an “ideal” faceted crystal) dendritic shape began to nucleate and grow (Figure 2k–n). Such perfect snowflake-like morphology [72] developed far more slowly, with a growth rate of 0.64 µm/s (Figure 2o). The six dendritic “trees” growing along the diagonal direction with significantly less, almost symmetrical, fractal-like branching, emphasize how close to perfection the sixfold symmetry was in this instance. 

Hybrid CH_3_NH_3_PbI_3–*x*_Cl*_x_* perovskite crystals displaying a sixfold symmetry and a rather compact dendritic structure reminiscent of simple star snowflakes [73] were further grown when a much lower rate of solvent evaporation was employed (Figure 2p–s). Thus, a growth rate of only 0.19 µm/s (Figure 2t) was generated when the solution was annealed at a lower temperature of 40 °C. Here, the branching of all six “trees” was much less prominent. The branching started to become more prominent along one of the six “trees” after a growing time of t_0_ + 150 s, when the crystal front along this particular “tree” was more prone to become favored by the nearby available perovskite molecules. Therefore, it developed faster by eliminating the competition from the other two “trees” in its vicinity, i.e., by achieving the possibility of more quickly accessing many more available perovskite molecules. 

The sixfold symmetry observed for all the aforementioned CH_3_NH_3_PbI_3–x_Cl_x_ perovskite crystals is a result of a predominant growth along the diagonal directions, with the growing tips closer to the reservoir of available perovskite molecules that could be “captured” and included in the crystal with a higher probability [74,75]. Moreover, the decrease in crystal growth rates observed with a decreased sample temperature can be explained by considering the finite amount of perovskite molecules available in the initial volume of 5 µL of solution and by assuming that at a lower processing temperature, the solvent evaporation rate is low and, consequently, induces only a minimal increase in the concentration of perovskite molecules over time. Thus, because in this case, the number of available molecules ready to attach to a crystal was low, the attachment probability was similarly low. Perovskite molecules had time to attach, detach and reattach until they reached an optimum position within the developing crystal, ultimately leading to more compact (i.e., less branched) crystals. On the other hand, using a high processing temperature causes a high rate of solvent evaporation, which, in turn, rapidly increases the concentration of perovskite molecules in the solution. Consequently, the number of available molecules ready to attach to a crystal “explodes”, along with the attachment probability. Therefore, perovskite molecules attach quickly within the growing crystal, leading to more branched perovskite crystals.

**Figure 3:**
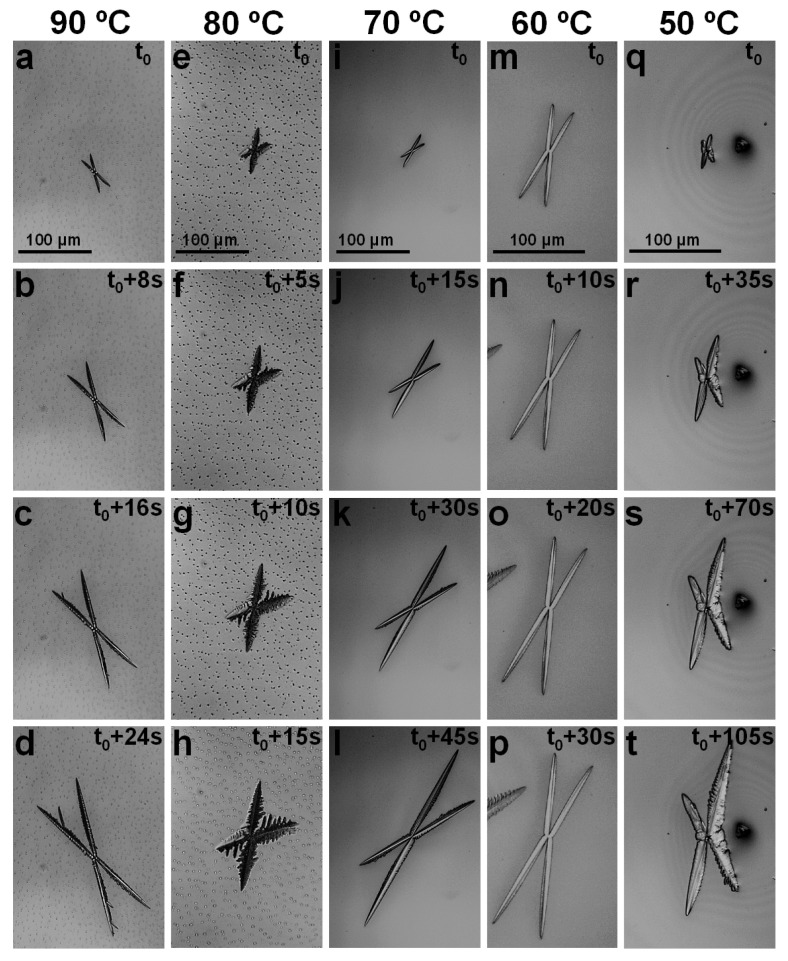
Optical micrographs depicting fourfold symmetrical crystals grown from hybrid lead-mixed halide CH_3_NH_3_PbI_3–x_Cl_x_ perovskite at processing temperatures of 90 °C (**a**–**d**), 80 °C (**e**–**h**), 70 °C (**i**–**l**), 60 °C (**m**–**p**) and 50 °C (**q**–**t**). Note that t_0_ is different for each temperature.

Interestingly, besides the perovskite crystals exhibiting a sixfold symmetry, other “X”-like crystals displaying a fourfold symmetry were also observed (Figure 3). This only occurred in the temperature processing range of 50 °C to 90 °C, and no “X”-like crystals were observed outside this range. While “X”-like crystals were observed to nucleate and grow independently of each other, their branching was rather limited, with more branching visible in crystals grown at 80 °C (Figure 3e–h). Furthermore, because the crystal branching seems to become more visible at later crystal growth stages, when, very often, crystals undergo a coalescence process, a direct correlation between the processing temperature and the degree of crystal branching is not yet possible to establish. Additionally, as determined from Figure 3, the crystal growth rates increased with the increase in processing temperature from ~2.24 µm/s (Figure 3q–t) to ~3.15 µm/s (Figure 3m–p), ~4.73 µm/s (Figure 3i–l), ~5.73 µm/s (Figure 3e–h) and ~6.69 µm/s (Figure 3a–d). These rates, despite the increased processing temperature, are much higher than the growth rates corresponding to the crystals exhibiting sixfold symmetry (shown in Figure 2e,j,o,t). For instance, while a fourfold “X”-like crystal grew at a rate of ~3.15 µm/s at 60 °C, a sixfold crystal grew at a rate of only 0.64 µm/s in the same sample and at the same temperature. Similarly, at 80 °C, the corresponding growth rates were ~5.73 µm/s and ~3 µm/s for the fourfold and sixfold symmetrical perovskite crystals, respectively. This suggests that the sixfold symmetrical crystals are of higher quality and could possibly exhibit puzzling optoelectronic properties, assuming their slower growth and the already demonstrated beneficial effects of the crystalline microstructure of mixed halide perovskites on their optical and photovoltaic properties [76]. To prove this tentative statement, future experiments are necessary to assess the internal structure of such crystals.

## 4. Conclusions

In this study, we grew crystals of an organic–inorganic halide perovskite precursor solution at different processing temperatures using an open-air setup that allowed us to change the solvent evaporation rate and to nucleate only a small number of crystals. We demonstrated that perovskite crystals grown at lower temperatures and, therefore, at lower solvent evaporation rates, displayed a clear sixfold symmetry with a shape reminiscent of rather compact dendrites. In contrast, six-branched crystals exhibiting a fractal dendritic morphology were obtained at higher temperatures. Fourfold symmetrical “X”-like crystals were also observed to nucleate and grow in the 50–90 °C temperature range but at higher growth rates as compared to their sixfold symmetry counterparts. This work is useful for providing model crystalline structures that can be employed in the future to correlate the optoelectronic properties of certain perovskites with their microstructure.

## Data Availability

The data presented in this study are available on request from the corresponding author.
